# Offensive or amusing? The study on the influence of brand-to-brand teasing on consumer engagement behavioral intention based on social media

**DOI:** 10.3389/fpsyg.2022.966254

**Published:** 2022-08-05

**Authors:** Yu-mei Ning, Chuan Hu, Ting-ting Tu, Dan Li

**Affiliations:** ^1^Business School, Yulin Normal University, Yulin, China; ^2^School of Business Administration, Zhongnan University of Economics and Law, Wuhan, China

**Keywords:** aggressive humor, brand dialogue, consumer engagement behavior, brand personality, social media

## Abstract

With the development of social media, advertising has migrated from traditional media to social media. Marketers are increasingly using social media’s brand pages to actively create humorous dialogue interactions with other brands for brand communication to achieve positive business outcomes. Especially brand-to-brand’s aggressive humor dialogue can also be an effective brand communication strategy. Based on benign violation theory, we have studied the influence mechanism and boundary condition of the brand-to-brand’s aggressive humor styles (low-aggressive and high-aggressive) on consumer engagement behavioral intention in social media context. Through experiments, it is indicated that low-aggressive humor could promote consumer engagement behavioral intention more than high-aggressive humor. Benign appraisal mediates the relationship between low-aggressive humor and consumer engagement behavioral intention. Furthermore, brand personality not only moderates the effect of low-aggressive humor on consumer engagement behavioral intention, but also moderates the mediating role of benign appraisal between low-aggressive humor and consumer engagement behavior intention.

## Introduction

With the popularity of the Internet, the number of users of social media continues to rise, and social media has taken up a large part of people’s daily life. Since social media can satisfy most people’s entertainment needs, “humor” has gradually become a prominent element of online communication on social media. Social media users are engaging with humorous content more than ever by spreading the humorous content to others through the network (Shifman, 2012). In addition to interpersonal interactions, brands also use playful entertainment content in anthropomorphic ways to promote consumer-brand interactions (Nielsen, 2015; Cheung et al., 2019). Among them, Durex and Burger King are typical examples of the successful use of anthropomorphic expressions to carry out humorous dialogues with other brands on social media. Durex@Wrigley Gum: “Honey, thank you for being on my left for so many years and being an excuse to buy me.” Burger King @ McDonald’s: “Every king needs a clown, happy 50th birthday!” Are brands using their brand pages on social media to actively create humorous dialogues and interactions with other brands in an ad that is attempting to engage consumers in the ad’s interactions, or is it just to entertain them? How effective are this brand’s humorous dialogues and interactions with other brands in motivating consumers to respond to what the brand is promoting in advertising?

Existing research on humorous communication has mainly explored the impact of humorous advertising (the humorous interaction between brands and consumers) on consumers. For example, many scholars have studied traditional media such as television and print media (Eisend, 2009; Warren et al., 2019) and online media (Dessart et al., 2015; Nair, 2016) humorous advertisements have a positive impact on consumers by breaking through the clutter of advertisements. The same is a humorous advertisement, and its humorous style will also be different, some funny and affiliative, and some witty and aggressive. The aggressive humorous posts and memes on social media are one of the most widely spread by users (Taecharungroj and Nueangjamnong, 2014). Research on aggressive humor advertising has pointed out that disparaging humorous television ads increase brand attitudes and advertising recall by increasing the superiority of those who seek high power (Newton et al., 2016). Gregory et al. (2019) argued that aggressive humorous television ads affect advertising effectiveness through individualistic culture’s perception of humor. However, only a few studies have organically combined aggressive humor and brand-to-brand interaction to explore the impact of brands’ aggressive humor dialogue interaction with other brands on consumers. Exploring this gap is important, as compared with traditional competing advertising, brand-to-brand dialogue is more likely to improve consumers’ perception of freedom and be more easily accepted by them (Thomas and Fowler, 2021). Meanwhile, aggressive humorous exchanges are desired by social media users and are commonly considered as the goal of some brand-to-brand interactions (Jargon, 2017), we combine aggressive humor and brand-to-brand interaction. Thomas and Fowler (2021) argued that witty brand teasing can be an effective brand communication strategy. Brand-to-brand low-aggressive humor (relative to high-aggressive humor) increases consumers’ interest in the brand initiating the dialogue by reducing consumers’ perceptions of manipulative intent. And the response to the dialogue brand needs to select the appropriate humor type according to the type of humor that initiated the dialogue brand, so that the communication effect will be better. This study explored the impact of low-aggressive humor from a negative cognitive perspective (i.e., perceptions of manipulative intent), ignoring the analysis of positive cognitive factors. Because this kind of aggressive competitive advertising will not only generate negative reviews from consumers, but also positive reviews. Even competing ads will have more positive reviews (e.g., perceived brand distinctiveness) (Berendt et al., 2018).

As the dominant theory of explaining the mechanism of aggressive humor’s effect, benign violation theory holds that humor occurs when a stimulus is evaluated as containing violation and also the violation is evaluated as benign. Individuals’ perception of violation and the degree of benignity will affect their perception of humor and their attitude toward the initiator of humor (Warren and McGraw, 2015). In this theoretical framework, aggressive humor may be considered a harmful offense, or it may trigger laughter, and whether the attack can be evaluated as benign directly determines whether the attack can trigger humor. The achievement of benign attack appraisal is affected by humor initiator and audience factors (Li and Wang, 2022). Meanwhile, consumers’ evaluation of brand-initiated humorous communication largely depends on the initiator (Romero and Cruthirds, 2006). Therefore, this research used the benign violation theory, based on social media context, with positive cognitive factors (i.e., benign appraisal) as the mediating variable, and the characteristics of the brand initiating the dialogue (i.e., brand personality) as the moderating variable, to study the influence of the brand’s aggressive humor style to other brands on consumer engagement behavioral intention, so as to provide implications for brand’s use of effective humor style on social media to bring brand communication and consumer engagement behavior.

## Theoretical review and basic hypothesis

### Humor and interpersonal humor style

Broadly speaking, humor includes any interesting communication that generates positive emotions and cognitions in individuals, groups, or organizations (Romero and Cruthirds, 2006). Humor has received increasing attention in organizational environment (Romero and Cruthirds, 2006) and advertising strategies (Eisend, 2009).

Importantly, humor is a double-edged sword. For this reason, Martin et al. (2003), for different forms of CEO humor, based on other-directed humor, divided into two styles: positive humor (affiliative humor) and negative humor (aggressive humor). (1) Affiliative humor refers to a form of benign humor that rewards others and aims to support others to enhance relationships with others. Praise is an example of affiliative humor; (2) aggressive humor refers to harmful humor forms that are entertained by belittling others. Teasing, whining, ridicule, and sarcasm are examples of aggressive humor (Martin et al., 2003). According to the level of aggression, aggressive humor can be divided into low-aggressive humor and high-aggressive humor (Wright and Roloff, 2013). For low-aggressive humor, the attack or disparagement component is less severe (or more lighthearted) (Speck, 1991), and in many cases the tension generated by disparaging another is a mixture of enjoyment tinged with anxiety or guilt over enjoyment of the disparagement (Beard, 2008). Thus, low-aggressive humor is described as prosocial teasing, including flirtatious teasing (Shapiro et al., 1991) and jocular mockery (Haugh, 2014). Conversely, high-aggressive humor relies on disparaging another and is a form of negative comedy designed to improve personal well-being by disparaging another person or group (Warren et al., 2018). High-aggressive humor meant dark, more acerbic in tone, and meant to wound rather than just amuse (Holbert et al., 2011). Thus, high-aggressive humor is described as antisocial teasing, including sarcasm (Ducharme, 1994), ridicule (Billig, 2005), and bullying (Smith et al., 2008).

### Brand-to-brand aggressive humor style

Since brands are like other users on social media, dialogue interactions can be conducted in front of actual or potential audiences in public. Therefore, through the anthropomorphic expression of the brand, consumers will regard the brand as a person, and use the cognitive method of interpersonal communication to form a perception of the brand (Kwak et al., 2015). Therefore, the concept of interpersonal humor proposed by existing research can be used for brand dialogue. For the purposes of the study, this research defined brand-to-brand low-aggressive humor (e.g., teasing) as: brand-generated content involving harmful humor directed at an identifiable target competing brand, having fun in a way that disparages the identifiable target competing brand, and the attack or disparagement component are less severe (or more lighthearted). Brand-to-brand high-aggressive humor (e.g., ridicule) is defined as: brand-generated content involving harmful humor directed at an identifiable target competing brand, having fun in a way that disparages the identifiable target competing brand, and the attack or disparagement component are severe (or more extreme). Since the inter-brand dialogue involves the subject brand of the dialogue interaction (the brand that initiates the dialogue) and the object brand of the dialogue interaction (the identifiable target competing brand), but compared with the object brand of the dialogue interaction, the subject brand of the dialogue interaction plays the leading role in the inter-brand dialogue, so that their use of humorous dialogue strategies has a greater effect on consumers (Romero and Cruthirds, 2006). Therefore, this study only focuses on the research of the subject brand of the dialogue interaction, that is, to explore how the humor style of the subject brand of the dialogue interaction affects consumers’ perception and behavioral intention of the subject brand of the dialogue interaction, but does not include research on consumers’ evaluation of the object brand of dialogue interaction.

### Aggressive humor type and consumer engagement behavioral intention

Consumer engagement in social media context encompasses affective, cognitive, and behavioral dimensions. Importantly, user engagement is defined as behavioral manifestations toward a brand which are expressions of underlying psychological states resulting from a consumer’s interactive relationship with a brand (Brodie et al., 2011). Consumer engagement is usually measured through likes, comments, and shares (Coelho et al., 2016). Likes are a way for consumers to react to brand-initiated posts by not only showing consumers endorsement of brand-initiated posts, but also giving them credibility. Comments are a way for consumers to provide their comments on posts by adding new content. Reviews can help companies maintain the conversation, but also give consumers the power to organize the conversation. Retweets are a channel for consumers to spread brand information. Consumers’ sharing expands the reach of brand information’s audience and enhances interactivity.

On social media, novel, smart and delightful content generates higher audience responses (Brennan et al., 2015). Funny, playful ads help develop relationships by promoting conversation with others, which in turn are better for consumers to share (Campo et al., 2013). Due to the aggressive characteristics of aggressive humor, it will evoke high arousal emotions (e.g., awe, surprise, anger, and anxiety) in the audience (Taecharungroj and Nueangjamnong, 2014), and many scholars found a strong relationship between high arousal emotions and virality (Kaplan and Haenlein, 2011; Berger and Milkman, 2012). Brand teasing or ridicule creates a sense of connection with audiences. Because audiences feel that the brand teasing or ridicule was created for their benefit, they respond by expressing appreciation. This appreciation can take the form of laughter or even involvement in the process of continuing to tease or ridicule.

In the social media context, brands have fun by disparaging identifiable target competing brands. When disparagement reaches extremes, as is the case of high-aggressive humor, it may be viewed as excessive to the point of hostility (Martin et al., 2003). Further, humorous advertisements that trigger negative emotional responses can lead to public complaints (Beard, 2008). Consumers may prefer other less offensive forms of humor (Speck, 1991). Therefore, high-aggressive humor may attenuate the positive effects of humor. In addition, Holbert et al. (2011) showed the potential divergent effects of low-aggressive and high-aggressive humor on persuasion in the context of political campaign. Thomas and Fowler (2021) argued that when brands use low-aggressive humor to dialogue with another brands, consumers show higher interest in the brand that initiated the dialogue. Thus, compared with high-aggressive humor, low-aggressive humor is more beneficial to increase consumers’ interest in the brand, and consumers’ intention to comment and share.

Therefore, we have put forward Hypothesis H1: Compared with high-aggressive humor, low-aggressive humor generates more consumer engagement behavioral intention.

### The mediating effect of benign appraisal

The benign violation theory is a theory that has been used in recent years to explain how humor occurs and affects its surroundings. The theory proposes that humor occurs when something that is perceived to threaten a person’s well-being, identity, or normative belief structure simultaneously seems okay, acceptable, non-threatening, harmless or inconsequential (i.e., benign appraisal) (McGraw and Warren, 2010; McGraw et al., 2012b). Individuals’ perceptions of violations and the degree of benignity will affect their perception of humor and their attitudes toward the humor initiator (Warren and McGraw, 2015). In other words, the perception that something that is wrong is actually okay can transform an otherwise negative experience to a positive experience characterized by laughter and amusement (Apter, 1982). Excessive violations of the norm, however, offend or threaten the perceiver, thereby inhibiting the effect of humor (McGraw and Warren, 2010). Norms are defined as perceptions, attitudes, and behaviors that a group or society approves of and abides by Baron et al. (1992). Violations include not only threats to physical well-being (e.g., tickling) but also identity threats (e.g., teasing, sarcasm) and behaviors that violate cultural norms (e.g., inappropriate dress), social norms (e.g., strange behavior), moral norms (e.g., disrespectful behavior), communication norms (e.g., puns, malapropisms) and logic norms (e.g., absurdities, non-sequiturs). Benign appraisal refers to an individual’s subjective perception of normative, acceptable, and sensible things, that is, a benign violation perception (McGraw and Warren, 2010).

Thus, according to the benign violation theory, brands disparaging identifiable target competing brands can be viewed by consumers as a violation of expected existing communication norms. Whether the disparagement can be evaluated as benign directly determines whether the disparagement can trigger humor. When brands attack or disparage the identifiable target competing brand less severely or more lightly (i.e., low-aggressive humor), the extent to which the normative violation of brand manipulation is perceived by consumers is relatively modest. However, when brands attack or disparage the identifiable target competing brand severely or to extremes (high-aggressive humor), the extent to which the normative violation of brand manipulation is perceived by consumers is relatively excessive. Therefore, compared with excessive norm violations, moderate norm violations are more likely to lead consumers to believe that the brand’s disparagement components against the identifiable target competing brand are acceptable and harmless, that is, it improves the benign appraisal of consumers, so it will be more humorous or amusing. Further, there is a link between appreciation of humor and the ability to interact effectively with others (Gervais and David, 2005). Simultaneously perceived humor can lead consumers to like advertisements and brands that initiate teasing in the advertisements (Eisend, 2011). Therefore, compared with high-aggressive humor, low-aggressive humor is more likely to form benign appraisal of consumers, which leads to more appreciation of humor by consumers, which further enhances consumers’ interest in the brand and improve consumers’ intention to comment and share.

Therefore, we have put forward Hypothesis H2: Benign appraisal plays a mediating effect between low-aggressive humor and consumer engagement behavioral intention; that is, compared with high-aggressive humor, low-aggressive humor generates more benign appraisal, which in turn leads to more consumer engagement behavioral intention.

### The moderating effect of brand personality

Benign violation theory also helps explain why similar experiences trigger laughter in some situations but not in others. For example, the same tickling that prompts laughter from a loved one (i.e., considered benign) won’t trigger laughter if the tickle is self-inflicted (no violation), nor will it trigger laughter if the tickler is a creepy stranger (i.e., seen as a threat and therefore not playful). In addition, previous research has demonstrated that consumers’ evaluation of brand-initiated humorous communication largely depends on the initiator (Romero and Cruthirds, 2006; Warren and McGraw, 2015). Accordingly, we investigate the effects of a boundary condition that seems especially crucial in the current context—that is, the personality of the brand. Brand personality, a set of human characteristics associated with brands, influences how observers perceive brand behavior and its use of humor (Sundar and Noseworthy, 2016). Indeed, it consists of five core dimensions (Aaker, 1997), of which sincerity and excitement are considered the most fundamental (Swaminathan et al., 2009). Sincere brands are viewed as being warm, authentic, consistent, and family oriented, while exciting brands are associated with youth, uniqueness, fun, and boldness, pushing boundaries (Aaker et al., 2004).

Aaker et al. (2004) pointed out that consumers should react unfavorably toward sincere brands that violate norms excessively. The benign violation theory argued that the playful nature of humorous aggression ads is likely to suggest to consumers that the teaser’s behavior is not overly threatening and that the attack is not serious or harmless (McGraw and Warren, 2010; McGraw et al., 2012a). Because sincere brands are associated with goodwill and authenticity, compared with excessive norm-violating high-aggressive humor, when sincere brands use low-aggressive humor, consumers are more likely to interpret the component of brands’ attack on the identifiable target competing brand as more appropriate and lightly (Aaker et al., 2004), and therefore is acceptable or harmless, that is, it increases consumers’ perception of norm-violating acceptable (i.e., benign appraisal), thus making consumers think that sincere brands are more amusing with low-aggressive humor, ultimately triggering more humor appreciation promotes more consumer engagement behavioral intention. Therefore, when the brand is sincere, low-aggressive humor can enhance consumers’ benign appraisal more than high-aggressive humor, and in turn, consumers will be more likely to interact with the brand on social media.

While the use of aggressive humor by exciting brands is somewhat protected from norm violations (Aaker et al., 2004). Therefore, when exciting brands use low-aggressive humor and high-aggressive humor, consumers are more likely to interpret the component of brands’ attack on the identifiable target competing brand as not overly threatening, and therefore not serious or harmless (Aaker et al., 2004; McGraw and Warren, 2010). In turn, it is easy to make the consumers think that it is amusing for exciting brands to use low-aggressive humor similar to high-aggressive humor, thus making no significant difference in consumer engagement behavioral intention.

Therefore, we have put forward Hypothesis H3: Brand personality moderates the mediating effect of benign appraisal between low-aggressive humor and consumer engagement behavioral intention; that is, H3a: when the brand is sincere, low-aggressive humor generates more consumers’ benign appraisal than high-aggressive humor, which in turn leads to more consumer engagement behavioral intention; H3b: When the brand is exciting, there is no significant difference in consumers’ benign appraisal of low-aggressive humor and high-aggressive humor, which in turn makes no significant difference in consumer engagement behavioral intention.

Furthermore, research has shown that when companies use humor on social media to address online public complaints, sincere brands are more likely to use affiliative humor and exciting brands are more likely to use aggressive humor, which in turn is more likely to make online audiences respond more positively (Béal and Grégoire, 2021). Thus, consumers will be more likely to evaluate a brand’s dialogue strategy positively when it matches the brand’s personality. Specifically, on the one hand, consumers will perceive that the use of less offensive low-aggressive by sincere brands related to intimacy and safety as a better match than the overly offensive high-aggressive humor, so it is easier to accept, which in turn can improve consumers’ evaluation of low-aggressive humor, and ultimately enhance consumers’ intention to engage. On the other hand, exciting brands are associated with youth and boldness, and audiences would expect exciting brands to be a bit provocative and frivolous (Béal and Grégoire, 2021). Thus, consumers will perceive that the use of low-aggressive humor and high-aggressive humor by exciting brands is matched, so that consumers have no significant difference in evaluation of low-aggressive humor and high-aggressive humor, and ultimately make consumers have no significant difference in consumer engagement behavioral intention.

Therefore, we have put forward Hypothesis H4: Brand personality moderates the effect of low-aggressive humor on consumer engagement behavioral intention; that is, H4a: When the brand is sincere, low-aggressive humor generates more consumer engagement behavioral intention than high-aggressive humor; H4b: When the brand is exciting, there is no significant difference in consumer engagement behavioral intention between low-aggressive humor and high-aggressive humor.

**FIGURE 1:**
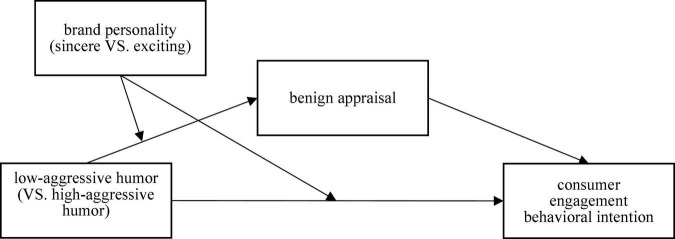
Theoretical model.

Based on the humorous style of brands attacking or disparaging the identifiable target competing brand, this research uses benign violation theory to construct a mediating path of “low-aggressive humor – benign appraisal – consumer engagement behavioral intention”; using brand personality to represent the characteristics of humorous dialogue initiators to explore its moderating effects on the mediating and the dependent variable, thus integrating all research variables to form the theoretical model of this research, as shown in Figure 1.

## Study 1: Testing the effect of low-aggressive humor (compared with high-aggressive humor) on consumer engagement behavioral intention

This study employs a single-factor (aggressive humor type: low-aggressive humor, low-aggressive humor), between-subjects experiment design to explore that low- aggressive humor (compared with high-aggressive humor) generates more consumer engagement behavioral intention, that is, hypothesis H1.

### Study design

#### Manipulation of aggressive humor type

In order to strengthen the external validity of the research results, this experiment is adapted from real brand attack cases in marketing practice. The experimental stimulus material selected the real mobile phone brand “Samsung.” Participants were first informed that Samsung and iPhone are two well-known competing mobile phone brands in China. In order to actively integrate into young consumer groups and better promote new products, Samsung posted on its official Sina Weibo a tweet for dialogue and interaction with its competing brands. Then, by designing different tweets to manipulate aggressive humor type, a total of two experimental materials for experimental conditions were formed. In the low-aggressive humor group, the tweet read: “You’re splashing in the bathtub @iphone, I’m taking a dip in the pool, doing what a man should do. Samsung Galaxy S8, IP68 super waterproof system.” In the high-aggressive humor group tweeted: “Apple with unreliable waterproof function, no matter how good-looking is, it is also fragile apple @iphone. Samsung specializes in water reversal, and it is more stylish and splash-proof. Samsung Galaxy S8, IP68 super waterproof system.”

#### Variable measurement

The items were all measured on a seven-point Likert scale (1 = strongly disagree at all, 7 = strongly agree), and the detailed measurement items are shown in Table 1. Referring to the literature of Pinkleton et al. (2002), three items consisting of ridiculing, mean-spirited, and negative of tweets were used to measure the participants’ perceptions of negativity of attack. Referring to the literature on aggressive humor by Speck (1991) and Cline et al. (2003), three items consisting of humorous, teasing, and sarcastic were used to measure participants’ perceptions of humor in brand tweets. The measurement of consumer engagement behavioral intention (α = 0.942) refers to the mature scale of Pentina et al. (2018). The sentence includes the following six items: I am likely to follow this brand; I am very interested in this brand; I would like this post; I am very likely to comment on this post; I am very likely to repost this post; I am very willing to repost this post. Finally, because the appreciation of humor varies from one individual to another, Warren et al. (2018) emphasized the importance of controlling the sense of humor, which we refer to the scale of Svebak (1996); second, it also controlled for the use of Sina Weibo (yes, no) and brand familiarity and brand favorability.

**TABLE 1:** Questionnaire items.

Construct	Item code	Item
Perceptions of negativity of attack	PNA1	I think this tweet is ridiculing
	PNA2	I think this tweet is mean-spirited
	PNA3	I think this tweet is negative
Perceptions of humor	PH1	I think this tweet is humorous
	PH2	I think this tweet is teasing
	PH3	I think this tweet is sarcastic
Perceptions of sincere	PS1	The brand could be described as sincere
	PS2	The brand could be described as warm
	PS3	The brand could be described as wholesome
	PS4	The brand could be described as family oriented
Perception of exciting	PE1	The brand could be described as exciting
	PE2	The brand could be described as unique
	PE3	The brand could be described as young
	PE4	The brand could be described as trendy
Benign appraisal	BA1	I think this tweet is not threatening
	BA2	I think this tweet is generally not a big problem
	BA3	I think this tweet is acceptable
	BA4	I think this tweet is reasonable
Perceptions of usefulness	PU1	This tweet helped me understand the product
	PU2	This tweet helped me with my purchase decision
	PU3	The tweet contained important product information
	PU4	This tweet provided me with useful product information
Perceptions of coolness	PC1	I think this tweet is cool
	PC2	I think this tweet is hip
	PC3	I think this tweet is trendy
	PC4	I think this tweet is stylish
Sense of humor	SH1	I easily recognize a hint, such as a wink or a slight change in emphasis, as a mark of humorous intent
	SH2	It is easy for me to find something comical, witty, or humorous in most situations
	SH3	I have much cause for amusement during an ordinary day
Use of Sina Weibo	USW1	Do you have a Sina Weibo account
Brand familiarity	BF1	How familiar are you with Samsung’s mobile phone brand
Brand favorability	BV1	How do you like Samsung’s mobile phone brand
Consumer engagement behavioral intention	CE1	I am likely to follow this brand
	CE2	I am very interested in this brand
	CE3	I would like this post
	CE4	I am very likely to comment on this post
	CE5	I am very likely to repost this post
	CE6	I am very willing to repost this post

#### Participants and study procedure

Based on the purpose of this research is the influence of the brand’s aggressive humor style on consumers, the participants in this study are consumers. To conduct study with human participants, this study received ethics approval from the Ethics Review Board of School of Business Administration of Zhongnan University of Economics and Law. All participants signed an informed consent form before they began the experiment.

This research context is social media platform, so the online survey is applicable, and as such, the participants were recruited online. We adopted the non-probabilistic convenience sampling method to collect data through professional online survey sites^1^. Respondents were provided CNY0.50 for their participation at the end of the survey.

Participants were randomly assigned to one of two experimental conditions and then read a tweet from a brand to promote a new product, and then asked participants to rate the brand’s engagement behavioral intention in the read tweets, and to provide their perceptions of negativity and perceptions of humor in brand dialogue. It also includes filling in brand familiarity, brand favorability, sense of humor, Sina Weibo usage, and relevant demographic information. In order to test whether the participants were attentive in the process of filling out the questionnaire, this experiment designed an attention test question. The item is “What brand did you evaluate in this survey?,” and the options are A. Lenovo, B. Coca-Cola, C. Samsung, D. BMW. If the participant’s choice is not option C, it is reasonable to think that the participant did not fill in the questionnaire carefully according to the experimental requirements, and it will be excluded as an invalid questionnaire. Finally, this experiment also designed a hypothesis guessing question. The item is “What is the hypothesis of this study?” In addition, we controlled the IP address to ensure that each volunteer participant only answered the questionnaire once. After data collection, we conducted strict screening and deleted the questionnaires with obvious regularity and too short response time. Among them, according to the average fastest reading of 500 words per minute by most people, the cases whose response time is less than 31 s were excluded.

### Study results

#### Manipulation testing

Excluding 32 people who failed the attention check, 46 people who guessed the hypothesis, and 112 people who answered the questions with obvious regularity and too short response time, the final sample was 620, of which 66.6% were male and 33.4% were female. The demographic breakdown of the sample is shown in Table 2 and the descriptive statistics for key constructs are shown in Table 3. All experimental measurement items loadings, AVE, and CR are included in Table 4. All experimental measurement model indices are included in Table 5. The results in Table 4 show that the measurement item loadings of all constructs exceed the reference value of 0.5, which supports convergent validity; The average variance extracted (AVE) values of all constructs exceeded the cutoff value of 0.50 and the AVE square root values are greater than inter-construct correlations (as shown in Table 6), which supports discriminant validity (Fornell and Larcker, 1981).

**TABLE 2:** Demographic breakdown.

Variable		Study 1		Study 2		Study 3	
		*N*	(%)	*N*	(%)	*N*	(%)
Gender	Male	413	66.6	317	51.1	178	55.6
	Female	207	33.4	303	48.9	142	44.4
	Total	620	100	620	100	320	100
Age	19 years old or below	18	2.9	33	5.3	13	4.1
	20–29 years old	359	57.9	356	57.4	194	60.6
	30–39 years old	209	33.7	211	34.0	97	30.3
	40–49 years old	31	5.0	17	2.7	12	3.8
	50 years old or above	3	0.5	3	0.5	4	1.3
	Total	620	100	620	100	320	100
Education background	high school or below	70	11.3	55	8.9	30	9.4
	College or bachelor	460	74.2	478	77.1	252	78.8
	Master or above	90	14.5	87	14.0	38	11.9
	Total	620	100	620	100	320	100
Use of Sina Weibo	Yes	590	95.2	596	96.1	310	96.9
	No	30	4.8	24	3.9	10	3.1
	Total	620	100	620	100	320	100

**TABLE 3:** Descriptive statistics.

Construct	Study 1		Study 2		Study 3	
	Low-aggressive humor	High-aggressive humor	Low-aggressive humor	High-aggressive humor	Low-aggressive humor	High-aggressive humor
Benign appraisal	–	–	M = 5.102	M = 4.522	M = 5.163	M = 4.470
	–	–	SD = 1.178	SD = 1.269	SD = 0.944	SD = 1.359
Consumer engagement behavioral intention	M = 5.441	M = 4.843	M = 5.054	M = 4.472	M = 5.174	M = 4.495
	SD = 1.187	SD = 1.416	SD = 1.454	SD = 1.222	SD = 1.091	SD = 1.364
Sense of humor	M = 5.556	M = 5.447	M = 5.298	M = 5.075	M = 5.404	M = 5.175
	SD = 0.978	SD = 0.968	SD = 1.088	SD = 1.046	SD = 0.915	SD = 1.074
Brand familiarity	M = 5.620	M = 5.400	–	–	–	–
	SD = 1.108	SD = 1.185	–	–	–	–
Brand favorability	M = 5.450	M = 5.030	–	–	–	–
	SD = 1.213	SD = 1.377	–	–	–	–

**TABLE 4:** Measurement model.

Construct	Item code	Item loadings	AVE	CR
**Perceptions of negativity of attack**				
	PNA1	0.803^a^/0.844^b^/0.874^c^	0.781^a^/ 0.712^b^/ 0.768^c^	0.914^a^/ 0.881^b^/ 0.908^c^
	PNA2	0.928^a^/0.797^b^/0.928^c^		
	PNA3	0.914^a^/0.888^b^/0.823^c^		
**Perceptions of sincere**				
	PS1	0.802^c^	0.504^c^	0.800^c^
	PS2	0.765^c^		
	PS3	0.640^c^		
	PS4	0.614^c^		
**Perception of exciting**				
	PE1	0.642^c^	0.564^c^	0.837^c^
	PE2	0.734^c^		
	PE3	0.794^c^		
	PE4	0.821^c^		
**Benign appraisal**				
	BA1	0.782^b/^0.721^c^	0.658^b^/ 0.623^c^	0.884^b^/ 0.867^c^
	BA2	0.746^b^/0.662^c^		
	BA3	0.861^b^/0.911^c^		
	BA4	0.849^b^/0.838^c^		
**Sense of humor**				
	SH1	0.782^a^/0.849^b^/0.760^c^	0.579^a^/ 0.614^b^/ 0.555^c^	0.805^a^/ 0.826^b^/ 0.789^c^
	SH2	0.710^a^/0.750^b^/0.737^c^		
	SH3	0.789^a^/0.747^b^/0.737^c^		
**Consumer engagement behavioral intention**				
	CE1	0.807^a^/0.806^b^/0.728^c^	0.728^a^/ 0.701^b^/ 0.661^c^	0.941^a^/ 0.933^b^/ 0.921^c^
	CE2	0.811^a^/0.780^b^/0.751^c^		
	CE3	0.855^a^/0.889^b^/0.844^c^		
	CE4	0.838^a^/0.794^b^/0.811^c^		
	CE5	0.905^a^/0.866^b^/0.877^c^		
	CE6	0.899^a^/0.881^b^/0.855^c^		

^a^Study 1 results.

^b^Study 2 results.

^c^Study 3 results.

**TABLE 5:** Measurement model indices.

Studies	Factors	Items	CFI	TLI	RASEA	SRMR	χ^2^	d.f.	*p*
2	4	16	0.947	0.934	0.079	0.050	468.89	96	<0.001
3	6	24	0.916	0.900	0.073	0.055	632.40	233	<0.001

**TABLE 6A:** Discriminant validity test of variables in study 1.

	1	2	3
1. Perceptions of negativity of attack	**0.884**		
2. Sense of humor	0.020	**0.761**	
3. Consumer engagement behavioral intention	−0.193**	0.468**	**0.853**

n = 620.

**p < 0.01.

The data marked in bold in the diagonal line of the matrix is the AVE square root, whereas the rest of the data is the corresponding correlation.

**TABLE 6B:** Discriminant validity test of variables in study 2.

	1	2	3	4
1. Perceptions of negativity of attack	**0.844**			
2. Benign appraisal	−0.115**	**0.811**		
3. Sense of humor	0.034	0.566**	**0.784**	
4. Consumer engagement behavioral intention	−0.154**	0.633**	0.533**	**0.837**

n = 620.

**p < 0.01.

The data marked in bold in the diagonal line of the matrix is the AVE square root, whereas the rest of the data is the corresponding correlation.

**TABLE 6C:** Discriminant validity test of variables in study 3.

	1	2	3	4	5	6
1. Perceptions of negativity of attack	**0.876**					
2. Perceptions of sincere	0.083	**0.710**				
3. Perception of exciting	−0.099	0.244**	**0.751**			
4. Benign appraisal	−0.331**	0.045	0.338**	**0.789**		
5. Sense of humor	0.040	0.344**	0.486**	0.327**	**0.745**	
6. Consumer engagement behavioral intention	−0.203**	0.191**	0.435**	0.667**	0.308**	**0.813**

n = 320.

**p < 0.01.

The data marked in bold in the diagonal line of the matrix is the AVE square root, whereas the rest of the data is the corresponding correlation.

The aggressive humor type manipulation test was successful. (a) Taking two experimental conditions (i.e., aggressive humor type) as the independent variable, and perceptions of negativity as the dependent variable, conduct paired-samples *t*-tests. The results showed that compared with the high-aggressive humor group (M = 5.353, SD = 0.928), the low-aggressive humor group (M = 3.082, SD = 1.533) perceived less negativity of attack [*t*(309) = −23.152, *P* < 0.01]; (b) The results of one-tailed *t*-tests respectively showed that the humor level of the low-aggressive humor group [M = 4.424, SD = 0.945, *t*(309) = 7.891, *P* < 0.01] and the high-aggressive humor group [M = 4.586, SD = 0.839, *t*(309) = 12.294, *P* < 0.01] was significantly greater than the mid-point (4) of the seven-point scale measuring humor.

#### Hypothesis testing

Taking aggressive humor type as the independent variable and the potential covariates as the dependent variable, a one-way MANOVA analysis was conducted. The results showed that there was a significant difference in brand familiarity [*F*(1,618) = 5.666, *P* = 0.018], brand favorability [*F*(1,618) = 16.191, *P* < 0.01], Sina Weibo usage [*F*(1,618) = 5.069, *P* = 0.025] and gender [*F*(1,618) = 7.974, *P* < 0.01] in the two aggressive humor types, and there was no significant difference in sense of humor, age and education background in the two aggressive humor types (all *P*s > 0.10). Then, taking aggressive humor type as the independent variable, consumer engagement behavioral intention as the dependent variable, and sense of humor, Weibo usage, brand familiarity, brand favorability, and demographic variables as the control variables, an ANCOVA analysis was conducted. The results showed that there was a significant difference in consumer engagement behavioral intention in the two aggressive humor types [*F*(1,611) = 17.426, *P* < 0.01]. Low-aggressive humor (M = 5.441, SD = 1.187) obtained more consumer engagement behavioral intention than high-aggressive humor (M = 4.843, SD = 1.416). Therefore, hypothesis H1 is verified.

Study 1 preliminarily verified that the low-aggressive humor group had a stronger engagement behavioral intention in social media than the high-aggressive humor control group. On this basis, the next study 2 will use brands of different product types to conduct experiments to further consolidate the results of study 1 and further explore the mediating mechanism of low-aggressive humor affecting consumer engagement behavioral intention.

## Study 2: Testing the mediating role of benign appraisal between low-aggressive humor and consumer engagement behavioral intention

On the basis of study 1, this study adopts a between-subjects experiment (low-aggressive humor vs. high-aggressive humor), the main purpose is to examine the mediating effect of benign appraisal between low-aggressive humor and consumer engagement behavioral intention, that is H2, thereby explaining why low-aggressive humor is more effective than high-aggressive humor. This study also re-examines the relative advantage of low-aggressive humor, by comparing the effect of low- aggressive humor and high-aggressive humor on consumer engagement behavioral intention, that is H1.

### Study design

The fictitious cake brand “TastyBakes” was chosen for the experimental stimulus material to avoid the interference of existing brands in the market and their brand image on the experimental results.

#### Manipulation of aggressive humor type

Referring to the experiment of Thomas and Fowler (2021), the experimental material is realized through the design of brand tweets. Participants were asked to imagine seeing a tweet from a consumer on Sina Weibo that read: “Just arrived abroad and need to buy a birthday cake for the party. How does @TopFrost compare to @TastyBakes?” TastyBakes and TopFrost are two competing cake brands in foreign countries. In order to better dialogue with consumers, promote consumers’ understanding of the brand and its products. TastyBakes responded to the consumer on its official Weibo account. We manipulated the aggressive humor type by designing different tweets, aggregated to form experimental material for two experimental conditions. In the low-aggressive humor group, the tweet read: “If you want frosting (in your tweets or in your cake), go to @TopFrost; if you want tasty food, go to @TastyBakes.” In the high aggressive humor group, the tweet read: “@TastyBakes food, always tasty, never frosty, please avoid @TopFrost.”

#### Variable measurement

The items were all measured on a seven-point Likert scale (1 = strongly disagree at all, 7 = strongly agree), and the detailed measurement items are shown in Table 1. The measures of perceptions of negativity of attack, perceptions of humor, and consumer engagement behavioral intention (α = 0.933) are consistent with study 1. The measurement of benign appraisal (α = 0.884) is adapted from previous studies by scholars (McGraw and Warren, 2010; McGraw et al., 2015; Warren and McGraw, 2015), and the measurement includes the following four items: I think this tweet is not threatening; I think this tweet is generally not a big problem; I think this tweet is acceptable; I think this tweet is reasonable. In addition, since previous research found that both information search (Java et al., 2007) and coolness attention (Toubia and Stephen, 2013) can motivate consumer engagement on Twitter, the two items of perceptions of usefulness and perceptions of coolness were added to exclude alternative explanations of consumer engagement behavioral intention. The perceptions of usefulness (α = 0.911) refers to the scale of Park and Lee (2009), which consists of four items. The perceptions of coolness (α = 0.929) refers to the scale of Rahman (2013), which consists of four items. Finally, sense of humor and Sina Weibo usage were also controlled.

#### Participants and study procedure

As the participants in this study are the same consumers as those in study 1, the ethics review was also conducted in this study and the informed consent was provided for the participants. As this study is an online survey as in study 1, this study also recruited participants through online survey sites (i.e., see text footnote 1). Respondents were provided CNY1.00 for their participation.

Manipulating the aggressive humor type, a questionnaire was pre-tested on 38 participants. After the pre-test was passed and the formal experiment was conducted, participants were randomly assigned to one of two experimental conditions and read a tweet from the brand in the experimental condition. Participants were then asked to rate the brand’s engagement behavioral intention in the read tweets, and to provide perceptions of negativity of attack and perceptions of humor in brand dialogue, and then to provide benign appraisal, perceptions of usefulness, perceptions of coolness. Also included are sense of humor, Sina Weibo usage, and relevant demographic information. Finally, the participants filled out the attention test question and the hypothesis guessing question. The attention test question is “What type of product does the brand in the reading material belong to?,” and the options are A. Car, B. Mobile phone, C. Food, D. Clothing. The hypothesis guessing question is the same as in study 1. In addition, this study controlled the IP address as in study 1. After data collection, consistent with study 1, this study deleted the questionnaires with obvious regularity and too short response time.

### Study results

#### Manipulation testing

Excluding 38 people who failed the attention check, 30 people who guessed the hypothesis, and 152 people who answered the questions with obvious regularity and too short response time, the final sample was 620, of which 51.1% were male and 48.9% were female. The demographic breakdown of the sample is shown in Table 2 and the descriptive statistics for key constructs are shown in Table 3. All experimental measurement items loadings, AVE, and CR are included in Table 4. The results in Tables 4–6 support the convergent and discriminant validity of all constructs.

The aggressive humor type manipulation test was successful. (a) The results of paired-samples *t*-tests showed that compared with the high-aggressive humor group (M = 4.846, SD = 1.022), the low-aggressive humor group (M = 3.475, SD = 1.622) perceived less negativity of attack [*t*(309) = −12.079, *P* < 0.01]; (b) The results of one-tailed *t*-tests respectively showed that the humor level of the low-aggressive humor group [M = 4.370, SD = 1.311, *t*(309) = 4.968, *P* < 0.01] and the high-aggressive humor group [M = 4.559, SD = 1.057, *t*(309) = 9.310, *P* < 0.01] was significantly greater than the mid-point (4) of the seven-point scale measuring humor.

#### Hypothesis testing

Taking aggressive humor type as the independent variable and the potential covariates as the dependent variable, a one-way MANOVA analysis was conducted. The results showed that there was a significant difference in sense of humor [*F*(1,618) = 6.741, *P* = 0.010] and gender [*F*(1,618) = 6.246, *P* = 0.013] and education background [*F*(1,618) = 5.619, *P* = 0.018] in the two aggressive humor types, and there was no significant difference in Sina Weibo usage and age in the two aggressive humor types (all *P*s > 0.10). Then, ① with aggressive humor type as the independent variable, consumer engagement behavioral intention as the dependent variable, and sense of humor, Weibo usage, and demographic variables as the control variables, an ANCOVA analysis was conducted. The results showed that there was a significant difference in consumer engagement behavioral intention in the two aggressive humor types [*F*(1,613) = 21.508, *P* < 0.01], and the low-aggressive humor (M = 5.054, SD = 1.454) obtained more consumer engagement behavioral intention than high-aggressive humor (M = 4.472, SD = 1.222). Therefore, H1 is verified again. ② With aggressive humor type as the independent variable, benign appraisal as the dependent variable, sense of humor, Weibo usage, and demographic variables as the control variables, an ANCOVA analysis was conducted. The results showed that there was a significant difference in benign appraisal in the two aggressive humor types [*F*(1,613) = 26.746, *P* < 0.01], and the low-aggressive humor (M = 5.102, SD = 1.178) obtained more benign appraisal than high-aggressive humor (M = 4.522, SD = 1.269). ③ With aggressive humor type as the independent variable, perceptions of usefulness and perceptions of coolness as the dependent variable, sense of humor, Weibo usage, and demographic variables as the control variables, an ANCOVA analysis was conducted. The results showed that there was no significant difference in perceptions of usefulness in the two aggressive humor types [*F*(1,613) = 2.667, *P* = 0.103]. There was no significant difference in perceptions of coolness in the two aggressive humor types [*F*(1,613) = 2.414, *P* = 0.121]. Thus, alternative explanations for perceptions of usefulness and perceptions of coolness are excluded.

#### Mediation effect testing

**FIGURE 2:**
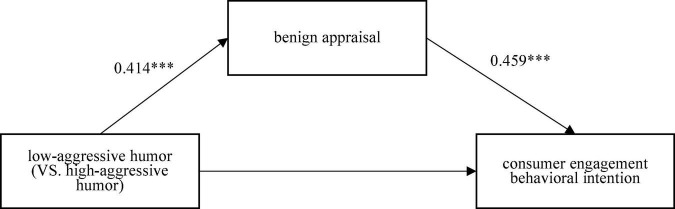
Path coefficient of low-aggressive humor on consumer engagement behavioral intention. Significance levels: *P* < 0.001 (^***^).

To test the mediating effect of benign appraisal between low-aggressive humor and consumer engagement behavioral intention, the aggressive humor type was set as a dummy variable (high-aggressive humor was coded as 0, low-aggressive humor was coded as 1), and sense of humor, Weibo usage, and demographic variables were used as the control variables. The maximum variance inflation factor (VIF) value in the models were 1.665, which was less than 3.3, indicating that variables did not contain problematic collinearity (Petter et al., 2007). As shown in the results of process model 4 (Figure 2), the mediating effect of benign appraisal was significant (indirect effect was β = 0.190, SE = 0.043; 95% CI = [0.091, 0.312]), that is, compared with high-aggressive humor, low-aggressive humor promoted consumer engagement behavioral intention by generating higher benign appraisal, which validates H2.

## Study 3: Testing the moderating effect of brand personality

This study is a 2 (aggressive humor type: low-aggressive vs. high-aggressive humor) × 2 (brand personality type: sincere vs. exciting) between-subjects experiment, the main purpose is to examine that brand personality moderates low-aggressive humor on consumer engagement behavioral intention, that is H3, and to examine brand personality moderates the mediating effect of benign appraisal between low-aggressive humor and consumer engagement behavioral intention, that is H4.

### Study design

#### Manipulation of aggressive humor type and brand personality type

Manipulating aggressive humor type through different tweets, manipulating brand personality types through different brands’ official Weibo banner images, company profiles, and brand logos and tweets, forming experimental materials for four experimental conditions in total. Following the procedure recommended by Aaker et al. (2004), first, brand personality was manipulated through vocabulary choice and phrasing. Use “hello” and “opponents” for sincere brands, and “hey” and “friends” for exciting brands. Use the hashtags “#family,” “#warm” and “#wholesome” for sincere brands and “#unique,” “#young” and “#trendy” for exciting brands. Second, the brand personality is manipulated by manipulating the banner image, company profile, and brand logo of the brand’s official Weibo page. Use an image of families for sincere brands, and an image of jumping young people for exciting brands. The company profile manipulation for sincere brand is: “Dear customer, we sincerely welcome you to Bigmeat. We are a #family#warm#wholesome fast food brand.” The company profile manipulation for exciting brands is: “Hey guy, we enthusiastically welcome you to Topburger. We are a #unique#young#trendy fast food brand.” Using blue font, the brand logo of Comic Sans font represents sincere brands; using orange font, the brand logo of **phosphate** font represents exciting brands. Therefore ① low-aggressive × exciting group: participants were asked to read a tweet of Topburger’ s aggressive humor to Bigmeat on Weibo that read: “Hey! @Bigmeat your cheeseburgers’ beef is freezing; our burgers’ beef is so fresh and too cool to ever be frozen. –From your friend @Topburger” ② low-aggressive × sincere group: participants were asked to read a tweet of Bigmeat’ s aggressive humor to Topburger on Weibo that read: “Hello! @Topburger your cheeseburgers’ beef is freezing; our burgers’ beef is so fresh and too cool to ever be frozen. – From your opponent @Bigmeat” ③ high-aggressive × exciting group: participants were asked to read a tweet of Topburger’ s aggressive humor to Bigmeat on Weibo that read: “Hey! Never thought your cheeseburgers are full of frozen beef @Bigmeat; our burgers’ beef is so fresh and so cool that it never be frozen. – From your friend @Topburger” ④ high-aggressive × sincere group: participants were asked to read a tweet of Bigmeat’ s aggressive humor to Topburger on Weibo that read: “Hello! Never thought your cheeseburgers are full of frozen beef @Topburger; our burgers’ beef is so fresh and so cool that it never frozen. – From your opponent @Bigmeat”

#### Variable measurement

The items were all measured on a seven-point Likert scale (1 = strongly disagree at all, 7 = strongly agree), and the detailed measurement items are shown in Table 1. The measures of perceptions of negativity of attack, perceptions of humor, benign appraisal (α = 0.865), and consumer engagement behavioral intention (α = 0.920) are consistent with study 2. Referring to the literature of Aaker et al. (2004) on perceptions of sincere and perception of exciting, four items consisting of sincere, warm, wholesome, and family oriented were used to measure participants’ perceptions of sincere; four items consisting of exciting, unique, young, and trendy were used to measure the participants’ perceptions of exciting.

#### Participants and study procedure

As the participants in this study are the same consumers as those in study 1, the ethics review was also conducted in this study and the informed consent was provided for the participants. As this study is an online survey as in study 1, this study also recruited participants through online survey sites (i.e., see text footnote 1). Respondents were provided CNY1.50 for their participation.

Participants were randomly assigned to one of the four experimental conditions, then read the company profile of the brand’s official Weibo page in the experimental condition, and then asked participants to provide perceptions of sincere and perceptions of exciting. Then read a tweet from the brand that had a dialogue and interaction with its competing brands for promotion, and then asked the participants to rate the brand’s engagement behavioral intention in the read tweets, and to provide perceptions of negativity of attack and perceptions of humor in brand dialogue, and fill in appraisal. Also included are sense of humor, Sina Weibo usage, and relevant demographic information. Finally, the participants filled in the attention test question and the hypothesis guessing question, which are the same as in study 2. In addition, this study controlled the IP address as in study 1. After data collection, consistent with study 1, this study deleted the questionnaires with obvious regularity and too short response time.

### Study results

#### Manipulation testing

Excluding 26 people who failed the attention check, 16 people who guessed the hypothesis, and 88 people who answered the questions with obvious regularity and too short response time, the final sample was 320, of which 55.6% were male and 44.4% were female. The demographic breakdown of the sample is shown in Table 2 and the descriptive statistics for key constructs are shown in Table 3. All experimental measurement items loadings, AVE, and CR are included in Table 4. The results in Tables 4–6 support the convergent and discriminant validity of all constructs.

The manipulation test was successful. ① (a) The results of between-subjects ANOVA showed that compared with the high-aggressive humor group (M = 4.235, SD = 1.535), the low-aggressive humor group (M = 3.590, SD = 1.674) perceived less negativity of attack [*F*(1,316) = 13.156, *P* < 0.01]; (b) The brand personality or the interaction between aggressive humor and brand personality did not have a significant effect on perceptions of negativity of attack (all *P*s > 0.10). ② The results of one-tailed *t*-tests respectively showed that the humor level of the low-aggressive humor group [M = 4.279, SD = 1.200, *t*(159) = 2.943, *P* < 0.01] and the high-aggressive humor group [M = 4.494, SD = 1.118, *t*(159) = 5.587, *P* < 0.01] was significantly greater than the mid-point (4) of the seven-point scale measuring humor. ③ The perceptions of sincere in the sincere brand group (M = 5.381, SD = 0.964) was significantly higher [*F*(1,316) = 43.908, *P* < 0.01] than that of the exciting brand group (M = 4.700, SD = 0.888). ④ The perceptions of exciting in the exciting brand group (M = 5.744, SD = 0.751) was significantly higher [*F*(1,316) = 124.913, *P* < 0.01] than that of the sincere brand group (M = 4.678, SD = 0.943). ⑤ The aggressive humor or the interaction between aggressive humor and brand personality did not have a significant effect on perceptions of brand personality (all *P*s > 0.10). ⑥ There was no correlation between the perceptions of sincere scale and the perceptions of exciting scale (*r* = −0.02, *P* > 0.10), indicating that these two brand personalities are exclusive, distinct, and independent (Aaker, 1997).

#### Hypothesis testing

**FIGURE 3:**
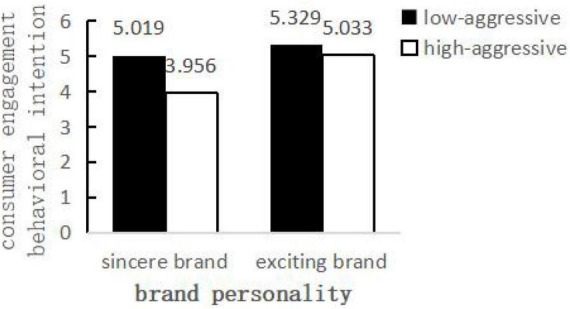
The influence of aggressive humor type and brand personality on consumer engagement behavioral intention.

Taking aggressive humor type and brand personality as the independent variable and the potential covariates as the dependent variable, a two-way MANOVA analysis was conducted. The results showed that there was no significant difference in sense of humor, Sina Weibo, and all demographic variables in four experimental conditions (all *P*s > 0.10). Then, ① with aggressive humor type and brand personality as the independent variables, and consumer engagement behavioral intention as the dependent variable, an ANOVA analysis was conducted. The results showed that (a) aggressive humor type had a significant effect on consumer engagement behavioral intention [*F*(1,316) = 26.823, *P* < 0.01]; (b) brand personality had a significant effect on consumer engagement behavioral intention [*F*(1,316) = 27.988, *P* < 0.01]; (c) Importantly, the interaction between aggressive humor and brand personality had a significant effect on consumer engagement behavioral intention [*F*(1,316) = 8.545, *P* < 0.01]. The simple effect analysis further showed (Figure 3) that in the sincere brand condition, low-aggressive humor (M = 5.019, SD = 1.134) generated more consumer engagement behavioral intention [*F*(1,316) = 32.824, *P* < 0.01] than high-aggressive humor (M = 3.956, SD = 1.311), which verified H4a; in the exciting brand condition, low-aggressive humor (M = 5.329, SD = 1.030) and high-aggressive humor (M = 5.033, SD = 1.200) was no significant difference in consumer engagement behavioral intention [*F*(1,316) = 2.545, *P* = 0.112], which verified H4b. ② Similarly, with aggressive humor type and brand personality as the independent variables, and benign appraisal as the dependent variable, an ANOVA analysis was conducted. The results showed that (a) aggressive humor type had a significant effect on benign appraisal [*F*(1,316) = 31.693, *P* < 0.01]; (b) brand personality had a significant effect on benign appraisal [*F*(1,316) = 35.220, *P* < 0.01]; (c) the interaction between aggressive humor type and brand personality had a significant effect on benign appraisal [*F*(1,316) = 8.617, *P* < 0.01]. The simple effect analysis further showed that in the sincere brand condition, low-aggressive humor (M = 4.978, SD = 1.005) generated more benign appraisal [*F*(1,316) = 36.681, *P* < 0.01] than high-aggressive humor (M = 3.925, SD = 1.312); in the exciting brand condition, low-aggressive humor (M = 5.347, SD = 0.845) and high-aggressive humor (M = 5.016, SD = 1.180) was no significant difference in benign appraisal [*F*(1,316) = 2.629, *P* > 0.01].

#### Mediation effect testing

Test the significance of the moderated-mediation model involving aggressive humor type as the independent variable, benign appraisal as the mediator variable, brand personality as the moderator variable, consumer engagement behavioral intention as the dependent variable, demographic variables, sense of humor, and Sina Weibo usage as control variables. The maximum variance inflation factor (VIF) value in the models were 3.123, which was less than 3.3, indicating that variables did not contain problematic collinearity (Petter et al., 2007). The process model 8 (moderated-mediation model) results showed that the mediating effect of benign appraisal was significant (β = 0.462, SE = 0.147; 95% CI [0.179, 0.756]). This indicates that the indirect effect of “aggressive humor type-benign appraisal-consumer engagement behavioral intention” is different depending on the brand personality conditions; specifically, this given sequence is significant for sincere brands (β = 0.582, SE = 0.130; 95%CI [0.336, 0.849]), which verified H3a; but not significant for exciting brands (β = 0.120, SE = 0.090; 95% CI [−0.053, 0.301]), which verified H3b.

## Discussion of the results

Through study 1 with mobile phone brands, we found that low-aggressive humor promotes consumer engagement behavioral intention more than high-aggressive humor. This conclusion is consistent with the research conclusions of scholars such as Thomas and Fowler (2021). On this basis, study 2 using cake brands to examine that low-aggressive humor can promote consumer engagement behavioral intention; benign appraisal play a mediating role between low-aggressive humor and consumer engagement behavioral intention. Distinguished from the study of Thomas and Fowler (2021), we explore the impact of low-aggressive humor from a positive cognitive perspective (benign appraisal) as opposed to negative cognitive factors (perceptions of manipulative intent). This result demonstrates the relationship between aggressive humor and consumer engagement behavioral intention from a new perspective. In addition, study 3 with the hamburger brand further compared the effect of two different aggressive humor on consumer engagement behavioral intention in two different brand personalities. It was found that sincere brands used low-aggressive humor to promote consumer engagement behavioral intention more than high-aggressive humor, consistent with the conclusion of previous study. Béal and Grégoire (2021) have proved that brand personality moderated the effect of humor type and likes/retweets/purchase intentions. Further, brand personality moderates the mediating effect of benign appraisal between low-aggressive humor and consumer engagement behavioral intention. Specifically, sincere brands used low-aggressive humor to increase consumers’ benign appraisal more than high-aggressive humor, and then it is easier to enhance the consumer engagement behavioral intention. This result is similar to that of the study of Béal and Grégoire (2021), that is, brand personality moderates the mediating effect of humor appreciation between humor type and likes/retweets/purchase intentions. These two results enrich the boundary condition of the relationship between brand-to-brand teasing and consumer engagement behavioral intention.

## Theoretical contributions and future research

### Theoretical contributions

(1) This research has enriched the connotation of humorous advertisements and the research on the influence effect of the interaction between brands. Although the impact of traditional non-brand anthropomorphic humor advertisements on consumers has been extensively studied, little research has examined the influence of brand anthropomorphic humorous advertisements on consumers (Eisend, 2009; Nair, 2016; Warren et al., 2019). This research enriches the connotation of humorous advertisements by exploring the influence of brand-to-brand teasing on consumer engagement behavioral intention. Moreover, existing research has mainly focused on how brand-consumer interaction affects consumers’ cognition (MacInnis and Folkes, 2017), but has not paid particular attention to how brand-brand interaction affects consumers’ cognition. This research extends the theory of interpersonal humor to the field of brand dialogue strategies, thereby dividing the aggressive dialogue styles that occur between brands on social media into two forms of humor, exploring the styles of brands’ aggressive dialogue in social media, and its influence on consumers’ cognition and behavioral intentions, which enriches the connotation of humorous advertisements and the research on the influence effect of the interaction between brands.

(2) This research has expanded the mechanism of the positive effect of low-aggressive humor between brands. A small number of existing studies on low-aggressive humor between brands have explored the effect of low-aggressive humor on brand interest through the role of negative cognitive factors such as perceptions of manipulative intent (Thomas and Fowler, 2021), while ignoring the role of positive evaluation factors. Based on benign violation theory, this research uses benign appraisal as a mediator to explore the mechanism of low-aggressive humor in promoting consumer engagement behavior from the perspective of positive evaluation, and enriches the application of benign violation theory in the field of inter-brand interaction research.

(3) This research has enriched the theory of brand personality. Although previous research on low-aggressive humor between brands focus on the moderating effect of the object brand factor of dialogue interaction (Thomas and Fowler, 2021), there has been very little examination of the moderating effect of humor initiator factors. Based on benign violation theory, consumers’ evaluation of brand-initiated humorous communication largely depends on the initiator (Romero and Cruthirds, 2006; Warren and McGraw, 2015; Béal and Grégoire, 2021). Therefore, from the perspective of the characteristics of the subject brand itself in dialogue interaction, this research explores the moderating effect of brand personality on the relationship between the brand’s low-aggressive humor and consumers’ engagement behavioral intention, thereby expanding the boundary condition that low-aggressive humor between brands brings positive effects also enriches the theory of brand personality.

### Managerial implications

Based on the social media context, this research studies the influence mechanism and boundary condition of brand-to-brand aggressive humor style on consumers’ engagement behavioral intention. The results of the study will bring inspiration to brands in terms of brand impression management, brand communication, and consumer behavior in the effective use of aggressive humor style on social media.

(1) On social media, brands can expand from focusing only on the brand-to-consumer relationship to also focusing on the brand-to-brand relationship. Even brands shouldn’t be afraid to engage in aggressive dialogue interaction with other brands, as brands creating aggressive humor dialogue with another brand can promote consumer engagement behavior. In particular, when brands use low-aggressive humor, it is more popular than high-aggressive humor. Therefore, marketers can actively use brand-teasing content strategies on social media brand pages to attract users on social media, thereby evoking user interaction with the brand.

(2) The dialogue between brands shapes consumers’ evaluation of brands and consumers’ engagement behavioral intention in social media. Brands should create content that obtains consumers’ benign appraisal. In particular, when a brand initiates an aggressive humor dialogue with another brand and uses low-aggressive humor, it can generate higher benign appraisal than high-aggressive humor. Therefore, brands should actively create low-aggressive humorous content that generates more benign appraisal.

(3) In social media context, brand teasing must also be in line with the brand’s personality in order to obtain consumers’ benign appraisal, and the brand should adjust its humor style according to the brand’s personality type. Exciting brands are more likely to use aggressive humor than sincere brands. Especially, compared with low-aggressive humor, exciting brands can also achieve good brand communication effects by using high-aggressive humor.

### Research limitations and future research

First, this research only collects humorous dialogues between mobile phone brands and retail food brands on social media, and future research needs to be extended to brands of other product types to improve the generalizability of the results. Second, this research only studies the humorous attacks that brands selectively target specific target brands, and frequently attacking or attacking too many target brands may damage the evaluation of the attacking brand (Kowalski, 2004), and moderators such as the frequency of brand attacks and the number of target brands of brand attacks need to be included for further discussion. Third, this research only selects brand dialogues on Sina Weibo social media, but the social norms of different social media are different. In the future, we can study whether the marketing effect brought by the aggressive humor style of other social media is consistent with the conclusions of this research. Fourth, due to the different understandings of humor in different cultures, humor tolerance, and normative beliefs, this research only considers the marketing effect brought by the brand aggressive humor style based on the Chinese context. In the future, we can explore the marketing effects of brand aggressive humor style on Western cultural backgrounds, individuals with different humor tolerance and individuals with different normative beliefs.

## Data Availability

The raw data supporting the conclusions of this article will be made available by the authors, without undue reservation.

## Footnotes

1
wjx.com
